# Enteric Methane Emissions in Dairy Cows with Different Genetic Groups in the Humid Tropics of Costa Rica

**DOI:** 10.3390/ani13040730

**Published:** 2023-02-17

**Authors:** Cristóbal Villanueva, Muhammad Ibrahim, Cristina Castillo

**Affiliations:** 1 Tropical Agricultural Research and Higher Education Center, Turrialba 30501, Costa Rica; 2 Animal Pathology Department, Veterinary School, University of Santiago de Compostela, Campus of Lugo, 27002 Lugo, Spain

**Keywords:** dry matter intake, crossbreds, emissions intensity, lactation stage, methane conversion factor, purebreds

## Abstract

**Simple Summary:**

Enteric methane is the main greenhouse gas araising from livestock production, which greatly contributes to global warming. A trial was carried out with dairy cows with different genetic backgrounds to determine the effect of genetics on the emission of enteric methane. Crossbred cows (50% *Bos taurus* × 50% *Bos indicus*) presented a lower annual emission of enteric methane compared to other cows with a greater European background. Crossbred cows had a greater adaptation to low tropical areas and a lower conversion of energy consumed to enteric methane. This knowledge contributes to the development of competitive farming with low carbon emissions.

**Abstract:**

Enteric methane (CH_4_) is one of the main greenhouse gases emitted in livestock production systems with ruminants. Among the options to reduce such emissions, animal genetics is one of the factors that is taking relevance in recent years. The aim of the present study was to assess the emission of enteric CH_4_ in dairy cows with different genetic backgrounds. Sixteen cows belonging to the following three genetic groups were selected for this study: seven F1 (50% Jersey × 50% Gyr), five Triple cross (50% Jersey × 31% Holstein × 19% Sahiwal) and four Jersey. Enteric CH_4_ emissions were measured in all cows for 15 months, at the middle of each month, using the SF_6_ technique. Enteric CH_4_ emissions did not differ (*p* > 0.05) among genetic groups, although it varied with the stage of lactation, due to differences in milk yield and dry matter intake (DMI). Pasture DMI and the intensity of CH_4_ emissions (g kg^−1^ DMI) differed (*p* < 0.05) between dry and lactating cows, with higher DMI in the lactation period, while CH_4_ emission intensity was higher for dry cows. Cows with the highest proportion of *Bos taurus* genes presented a higher annual mean methane conversion factor (Y_m_), with 7.22, 7.05 and 5.90% for the Triple cross, purebred Jersey and F1, respectively. In conclusion, non-significant differences in enteric CH_4_ emissions and Y_m_ were detected among dairy cows with different genetic backgrounds. However, F1 cows tended to show lower enteric CH_4_ emission and Y_m_, compared to those with more *Bos taurus* genes.

## 1. Introduction

Livestock production in Central America is a relevant activity, covering more than 30% of the land, with approximately 13 million head of cattle [1], and represents the main means of life for more than 0.5 million families throughout the cattle production chain. Livestock farming plays an important role in the economy of the Central American countries, as it contributes between 8 and 38% of the Agricultural Gross Domestic Product [2]. However, in recent years, livestock farming has been associated with a series of negative environmental impacts, such as deforestation [3], soil degradation, water pollution and the reduction of biodiversity [4]. Those effects are due to poor management inefficiency of livestock farms, which makes them less resilient to climate change and more prone to produce higher greenhouse gas emissions.

At the global level, livestock activities contribute to an average of 14.5% of total greenhouse gas (GHG) emissions considering all emissions throughout the production chain [5]. Methane emissions in livestock systems are mainly due to the following sources: (i) CH_4_ emissions from enteric fermentation; (ii) CH_4_ and nitrous oxide (N_2_O) emissions from manure management; (iii) direct emissions from synthetic nitrogen fertilizers; (iv) carbon dioxide (CO_2_) emissions from the use of fossil fuels due to the use of agricultural machinery and equipment on the farm; and (v) CO_2_ emissions from land use changes [6].

In Costa Rica, GHG emissions have reached 10,881.68 Mg CO_2_e, of which 22.47% correspond to livestock activities [7]. The main source of GHG emissions in Costa Rican cattle farms with different production systems and under diverse agroecological zones is enteric CH_4_, representing between 69 and 82% of total emissions [8,9,10]. Enteric CH_4_ represents an inefficiency in the use of energy, which in general, represents between 6 and 10% loss of total gross energy consumed. In dairy cows, energy losses through enteric CH_4_ emissions are comparable with the loss of 25–40 effective grazing days per year [11].

Previous studies indicate that genetic backgrounds have an influence on enteric CH_4_ emissions, which might be associated with the live animal weight and level of milk production. Dos Santos Pedreira et al. [12] found that Holstein cows present higher enteric CH_4_ emissions than Holstein × Zebu crossbreds (419 vs. 376 g CH_4_ cow^−1^ day^−1^). There is currently much interest in further studies to identify individuals within the breeds with functional characteristics for lowering enteric CH_4_ emissions. In addition, other studies are focused on traits with the potential for predicting CH_4_ emissions, such as feed intake, feed efficiency (residual feed intake) and energy balance [13]. However, in other trials, concentrate supplementation to cows grazing high-quality species improves milk production and quality and reduces the intensity of enteric CH_4_ emissions [14,15]. Other options to reduce enteric CH_4_ emissions have been evaluated, for example, the inclusion of lipids in the diet and the use of nitrates, ionophores, tannins and alkaline treatments [6]. The most recent studies have found that the use of 3-nitrooxypropanol reduces enteric CH_4_ emissions by 30 to 90% with no side effects on the milk yield [16,17,18,19]. A similar trend has been found with the use of red algae (*Asparagopsis* sp.) with reductions in CH_4_ emissions of 40 to 90% and improvements in feed conversion [20,21,22]. The decision to implement a given strategy implies the consideration of key criteria in the production systems, such as the interaction between mitigation potential and productivity [6].

In the Central American region, highland dairy farms (at an altitude >1000 m above sea level) predominantly use breeds such as Holstein and Jersey. The first is for its higher milk production, while the second is for its milk quality (high fat content and total solids) and better adaptation on land with steep slopes [23]. In lowlands, farmers have had a greater preference for *B. indicus* × *B. taurus* dairy cows in different proportions of both genetic groups [24]. Among the *B. indicus* breeds most used in crosses are the Gyr and Brahman. This crossing ensures greater adaptation to heat stress conditions [24,25].

The objective of this study was to determine the enteric CH_4_ emissions in pure-bred (*Bos taurus*) and cross-bred cows (*B. taurus* × *B. indicus*), during different stages of lactation and in the dry period, managed under grazing in the humid tropics of Costa Rica.

## 2. Materials and Methods

### 2.1. Trial Farm

This study was carried out at CATIE dairy farm, which is located in an area classified as a Very Humid Premontane Forest Life Zone [26], at an altitude of 600 m above sea level. In the research site, the temperature, annual rainfall and relative humidity were 22.9 °C, 2600 mm and 87.7%, respectively, as an average for the last five years. The study was conducted for a period of 15 months between 2016 and 2017. All procedures were accomplished in accordance with the mandatory regulations of Animal Welfare approved by the Congress of Costa Rica on 19 January 1998 (see https://www.mep.go.cr/sites/default/files/page/adjuntos/ley-no-7451-bienestar-animal.pdf, accessed on 7 January 2023).

### 2.2. Cow Selection and Feeding

Sixteen cows in the first stage of lactation (< than 100 days), belonging to the following breed groups were chosen: 7 F1 (50% Jersey × 50% Gyr), 5 Triple cross (50% Jersey × 31% Holstein × 19% Sahiwal) and 4 Jersey cows. Those cows were managed separately from the rest of the herd to prevent equipment damages. Those cows rotationally grazed on *Megathyrsus maximus* cv. Mombasa paddocks, with a one-day occupation period and 30 days of resting. They were also supplemented with a commercial concentrate made of soybean meal, citrus pulp (a byproduct of the orange industry), sugarcane molasses and elephant grass (*Pennisetum purpureum* Schum) cv. Taiwan (Table 1).

The dry matter intake of the above-mentioned feed is presented in Table 2. The intake of the grass *M. maximus* cv. Mombasa managed under grazing was estimated using the chromic oxide technique [27,28]. The commercial concentrate was offered based on the level of milk yield at a 1:3 ratio (1 kg of concentrate per 3 kg of milk). The amount of other feeds consumed was estimated as the difference between the amount offered and rejected. The amounts offered for feeds different from concentrates were similar for all cows, following the farm’s feeding plan.

### 2.3. Measurement of Enteric Methane Emissions and Other Variables

Enteric CH_4_ was quantified by using the sulfur hexafluoride (SF_6_) protocol [29], as adjusted for its use in tropical regions by Berndt et al. [30]. Before starting experimental measurements, the following activities were carried out as part of the technique:

- The calibration of the emission rate of the tracer gas (SF_6_) in the permeation tubes (capsules) was made by placing the tubes in an incubator at 39 °C for 12 weeks and weighed two times per week. All tubes presented a tracer gas emission curve with an R^2^ of 0.999, and those with lower values were rejected. The emission rate data were also used to estimate the life span of the tube and to calculate the daily emission of enteric CH_4_ per cow. Tubes registered a daily emission that varied between 3.36 and 5.05 mg day^−1^ (average = 4.16 ± 0.07 mg day^−1^).
- Two weeks before starting measurements, tubes were placed into the rumen using a cannula to ensure placement, in the same way that any bolus is administered.
- Two weeks before starting the trial, cows were adapted to carry a halter and a canister around the neck.
- The sampling line was adjusted to identify the critical points where they could get broken or uncoupled with cows’ movements. These pieces were adjusted, and damages were reduced after making adjustments. One method used for reducing damages was to manage the cows separately in the feeding and milking parlor.

Enteric CH_4_ measurements in each cow were made once a month for 15 months. Cows carried the measurement equipment for a period of 24 h each time. At the end of such period, canisters not having a pressure between 500 and 700 millibars were discarded. Moreover, two sampling lines were used as a control, for the monitoring of SF_6_ and CH_4_ in the environment of the paddocks, placing the equipment at 2 m height above ground, based on Berndt et al. [30].

Samples were sent to the laboratory of the National Institute for Innovation and Transfer of Agricultural Technology (INTA) of Costa Rica. Methane analysis was performed using a gas chromatographer (Agilent 7890A model) that uses flame ionization detectors (FIDs) and carbon capture electrons (ECDs). The amount of enteric CH_4_ per cow (g cow^−1^ day^−1^) was calculated by using the following formula, as suggested by Berndt et al. [30]:

CH_4_ (g day^−1^) = SF_6_ TP × (CH_4_ canister − CH_4_ environment)/(SF_6_ canister − SF_6_ environment)

where:

SF_6_ TP = permeation rate of SF_6_ from the tube (mg day^−1^)

Methane daily emission per cow was calculated in the dry period, as well as at different stages of the lactation period (<76 days, 76–150 days and >150 days). These stages were defined accordingly to the average behavior of the lactation curves of the cows in CATIE’s dairy farm as registered in the VAMPP Bovine 3.0 registry system (http://www.vampp-cr.com/, accessed on 5 February 2022). Furthermore, the annual emission of CH_4_ per cow was estimated, considering the weighted emission during the three samplings within the lactation period (280 days) and the one in the dry period (85 days). The length of the dry period was the average established based on Costa Rican statistics for intensively managed dairy farms [31].

Other variables were also measured, such as cow’s body weight, daily milk production, DMI of grasses consumed from the paddocks, the cut and carry forages and concentrates offered along the trial. The above-mentioned variables were used to determine the emission intensity based on milk production and DMI. In addition, the CH_4_ conversion factor (enteric CH_4_ energy as a percent of gross energy intake) and the Y_m_ per cow were estimated.

### 2.4. Statistical Analysis

Variables, such as daily emission of enteric CH_4_, milk production and emission intensity per kilogram of milk and per kilogram of dry matter intake, were analyzed by using analysis of variance, after testing the assumptions of normality and homogeneity of variances. As the errors presented a non-normal distribution, a mixed generalized linear model with Gamma distribution and logarithmic link function was used. Breed groups and the covariates of days of lactation and live weight of the animal at the time of sampling were considered fixed effects, whereas animals were considered random effects. Fisher’s LSD test was applied for the comparison of treatment means (*p* < 0.05) [32].

The analysis of variance was performed using general and mixed linear models to evaluate the effect of the breed and lactation period on the Y_m_ variable. The structure of treatments was given by the combination of the genetic group factors (with three levels: F1, Triple cross and Jersey) and lactation stages (with four levels: dry, <76 days, 76–150 and >150 days). The model included fixed effects of the factors breed and lactation stage and their interaction, the DMI covariate and the random effect of each animal. Due to the presence of heterogeneity of variances between stages of lactation, the structure of the matrix of variances and covariances was modeled considering a different variance for each level of the period. For the selection of the best model, the Bayesian (BIC) and Akaike (AIC) information criteria were used. Treatment means were evaluated using the Di Rienzo, Guzmán y Casanoves (DGC) mean comparison test (*p* < 0.05). Statistical analyses were performed using Infostat software [32].

## 3. Results

### 3.1. Enteric Methane Emission in Dairy Cows by Genetic Group

No significant differences (*p* > 0.05) in the daily enteric CH_4_ emissions for breed groups were detected at the different physiological stages (dry and three lactation periods). Dry cows had the lowest enteric CH_4_ emission, and during lactation, the lowest values corresponded to late lactation (>150 days). Cows with a higher proportion of *B. taurus* genes tend to present higher enteric CH_4_ emissions (Table 3). The standard error of the data indicates that there were relatively large variations between cows of the same genetic group, and therefore, a larger number of animals will be needed in future trials.

Regarding to the annual emission of enteric CH_4_, no significant (*p* > 0.05) differences were detected between breed groups. However, between 80 and 81% of the annual enteric CH_4_ emissions were produced during lactation. The total annual enteric CH_4_ emission varied between 91 and 111 kg cow^−1^, with the F1 cows having the lowest emission value (Table 4).

### 3.2. Feed Consumption, Milk Production and Intensity of Enteric Methane Emissions

Genetic groups had no effect (*p* > 0.05) on dry matter intake and milk production, as well as on the intensity of emissions per unit of milk produced and unit of dry matter consumed. Differences between non-lactating cows and the ones in different lactation stages were detected (*p* < 0.05) for total grass dry matter intake and the intensity of CH_4_ emissions per unit of dry matter consumed. Dry matter intake was higher during lactation, whereas the enteric CH_4_ emissions per unit of dry matter intake were higher for dry cows, as compared to those in lactating cows (Table 5).

### 3.3. Enteric CH_4_ Conversion Factor of Grazing Cows with Different Genetic Groups

Significant differences (*p* < 0.05) between breed groups were detected for the enteric CH_4_ conversion factor (Y_m_) during the dry period, but not for the lactation stages (*p* > 0.05; Table 6). Dry cows showed a higher Y_m_, the meaning of which can be related to the quality of the diet. The enteric CH_4_ conversion factor varied with lactation stages, with lower values for cows of less than 76 days, increased in those of 76–150 days, and tending to decline in the final stage of lactation (>150 days). For the annual average, cows with the highest proportion of *Bos taurus* genes presented a higher Y_m_.

## 4. Discussion

### 4.1. Enteric CH_4_ Emissions in Dairy Cows

There was no evident effect of the genetic group on daily enteric CH_4_ emissions; however, cows with more *Bos taurus* genes (Jersey) showed the highest values in both non-lactating (dry period) and lactating stages. This trend could be explained by the higher CH_4_ conversion factor obtained for a such genetic group, as shown in Table 6. Dos Santos Pedreira et al. [12] found a similar pattern in a study with purebred Holstein cows and Holstein × Gyr (F1) cows, in which purebred cows had higher enteric CH_4_ emissions in both dry and lactating periods. Dry cows emitted 261 and 238 g of enteric CH_4_ day^−1^, while in the lactation period, the corresponding values were 403 and 296 g day ^−1^, for the Holstein and F1 cows, respectively. Purebred cows doubled the milk production yield of F1, and this influenced the greater difference in enteric CH_4_ emission.

Different studies have found that enteric CH_4_ emissions are related to dry matter intake, which presents the highest values in early lactation when the highest milk yield is achieved [33,34,35]. There is evidence that residual intake is an indirect means to reduce enteric CH_4_ emissions, due to reduced intake and improved feed conversion. This functional condition of cattle can reduce enteric CH_4_ emissions by 15–25% and has moderate heritability and repeatability in dairy and beef cattle [36]. Currently, there is much interest in identifying individuals within breeds with the functional characteristic of lower enteric CH_4_ emission. In addition, other traits with potential for predicting CH_4_ emissions are feed intake, feed efficiency (residual feed intake) and energy balance [13].

The annual emission of enteric CH_4_ varied between 91 and 112 kg cow^−1^, being 22% higher in cows with a higher proportion of *Bos taurus* genes. This trend could be explained by higher dry matter intake values. Methane emissions found in this study were higher than those recommended by the IPCC [37] at Tier 1 (default value) for lactating cows of the Latin American and Caribbean region, which is 63 kg animal ^−1^·year^−1^. The highest CH_4_ emissions and milk yield per cow found in the present study are similar to those recommended for cows producing 6000 kg year^−1^ in Western Europe. Emission results showed a significant gap between those found with Tier 2 and Tier 1. Wilkes et al. [38] argued that if countries want to monitor the relationship between productivity and GHG emissions with the improvements implemented in the farms, the use of levels 2 or 3 is required. This means that there is a need for generating local emission factors or for establishing an information management system in representative farms of the sector to apply the IPCC [37] Tier 2 recommended values.

### 4.2. Enteric Methane Emission Intensity

Although no significant statistical differences were detected for the intensity of emissions per kilogram of milk produced, this tended to be reduced by 7 and 14% as the *B. taurus* genes declined in the Triple cross and F1 cows, respectively. Such little difference is due to the low values of enteric CH_4_ emissions observed in the F1 cows as compared to those obtained for the breed groups with more *Bos taurus* genes. Milk production per lactation for the three breed groups was similar, at 5041, 5055 and 5313 kg for the Jersey, F1 and Triple cross breed groups, respectively.

In other studies, the CH_4_ emission intensity was higher than that obtained in this study. For example, Muñoz et al. [34], working with Holstein Friesian cows producing less than 15 kg day^−1^ in late lactation (253 ± 18 days), obtained values between 34 and 36 g CH_4_ kg^−1^ of milk. A similar situation was found by Van Wyngaard et al. [14], who obtained values varying between 21.1 and 35.5 g CH_4_ kg^−1^ in Jersey cows, producing between 9.1 and 18.2 kg milk day^−1^ at 99 ± 18 days of lactation.

The intensity of emissions is an indicator of production efficiency, which reflects the maximization of the energy consumed by cattle into milk or meat production or a lower conversion factor of gross energy to enteric CH_4_. Poore and Nemececk [39] reported that there are communities interested in recognizing the efforts of producers offering products with a neutral or positive carbon footprint. It is expected that in the short and mid-term, this criterion would become a determinant for livestock products to achieve a better position in the market, in terms of acceptance by consumers.

Nevertheless, there are studies showing that a lower intensity of emissions per product does not guarantee a reduction in global warming or in the contribution to the absolute emission reduction goal. Sharma [40] reported that between 2005 and 2015, at the global level, there was an 11% reduction in CH_4_ emissions per liter of milk produced, but the absolute emissions increased by 18%. This is because the big dairy companies in the world increased their operations, as well as the number of animals. This situation does not reflect a positive impact of the livestock sector on the reduction of GHG emissions. Hence, the absolute reduction of CH_4_ and other GHGs implies a series of challenges for the livestock sector, such as to promote the responsible consumption of animal food sources and sustainable intensification for achieving greater production per unit of land. The latter could lead to a reduction in herd size and the area devoted to livestock or at least to maintaining the current area. Cassandro et al. [41] and Garnsworthy [42] argue that farms have the potential to optimize the herd structure for greater profitability and lower enteric CH_4_ emissions, through improvements to factors, such as milk yield per cow, energy use efficiency, reproductive parameters and the management of only the necessary cow’s replacements in the farm.

When we refer to new products offered to the consumers, Brazil is the world pioneer in launching carbon-neutral meat to the market. To achieve this goal, livestock farms established silvopastoral or agro-silvopastoral systems, where the predominant species are *Urochloa brizantha* grass and timber species, such as *Eucalyptus* sp. [43]. Currently, other countries, such as Australia and New Zealand, have launched a plan to achieve carbon neutral livestock products in 2030 and 2050, respectively. In both cases, the main strategy is the management of tree cover to offset GHG emissions [44,45]. Moreover, those countries are recognizing the importance of the carbon footprint as part of the marketing strategy for sustainable food systems.

### 4.3. Enteric CH_4_ Conversion Factor of Grazing Cows with Different Genetic Groups

In general, during the lactation period, the different breed groups showed a higher Y_m_ in the first two thirds of lactation, which decreased in the final third. It is likely that this trend was related to a higher DMI occurring in the first two thirds of lactation. However, for the breed groups, the Y_m_ was higher in non-lactating than in lactating cows, which could be related to the better quality of feeds offered to cows during the lactation period. Muñoz et al. [34] and Montenegro et al. [35] found lower Y_m_ values (6.2–8.1 and 6.6–7.5) in cows receiving higher amounts of concentrates. The lower values obtained by Montenegro et al. [35] might have also been influenced by the lower neutral detergent fiber content of the concentrates in the second study.

The Y_m_ adjusted per year was different between breeds groups; F1 cows presented a value 19 and 22% lower than that for the Jersey and Triple crossbred cows, respectively. The lowest Y_m_ value (6.5) obtained for the F1 cows was 10% lower than the one proposed by the IPCC [37] for dairy cattle. In contrast, the Jersey and Triple-crossbred groups showed Y_m_ values of 7.05 and 7.22, respectively, which are slightly higher than the IPCC reference value.

This means that the Y_m_ value must be adjusted according to the lactation stage, the genetic makeup and the quality of the diet fed to cows. Likewise, Montanholi et al. [46] argue that in tropical regions, *Bos taurus* cattle will be exposed to greater heat stress, resulting in lower cortisol secretion, which reduces cattle metabolic efficiency. This means that under stress conditions, there is higher residual feed consumption, which consequently increases the emission of enteric CH_4_.

In the Central American region, farmers dedicated to milk production in the lowlands use cows with *B. taurus* × *B. indicus* genetics. With this type of crossing, they have achieved an animal with greater adaptation to heat stress as a result of climate change [24]. The results of this study constitute local emission factors with the potential to be used in national GHG inventories and thereby achieve data that are more adjusted to the reality of the region’s production systems [38]. In addition, they are input for the formulation of policies, design of financial mechanisms and for markets where the effort of cattle producers with low carbon emissions is valued.

## 5. Conclusions

The results showed that there is no significant differences in enteric CH_4_ emissions and Y_m_ among dairy cows with different genetic backgrounds. However, F1 cows tended to show lower enteric CH_4_ emission and Y_m_, compared to those with more *Bos taurus* genes, and the integration of these genetic groups of animals in cattle production systems in the tropical regions might result in greater resilience to climate change and better opportunities for improving competitiveness of the systems.

## Figures and Tables

**Table 1:** Dry matter (DM) content and chemical composition of the supplements used in cows.

Feed	Dry Matter (%)	Crude Protein (%)	Neutral Detergent Fiber (%)	Digestible Energy (Mcal kg DM^−1^)
Concentrate	87.00	18.98	13	3.50
Soya flour	88.00	47.75	11	3.30
Citrus pulp ^1^	87.00	4.00	25	2.85
Sugarcane Molasses	72.60	3.80	0	3.05
*M. maximus* cv. Mombasa	16.10	11.10	57.60	1.90
*P. purpureum* cv. Taiwan	20.60	7.00	69.00	2.10

^1^ By-product of the orange industry.

**Table 2:** Daily feed intake (kg DM cow ^−1^) by lactating and dry (non-lactating) cows.

Food	Lactating Cows	Dry Cows
Concentrate	5.55	0.46
Soy flour	0.36	-
Citropulp ^1^	1.95	0.31
Molasses	0.52	0.08
*M. maximus* cv. Mombasa	6.87	7.9
*P. purpureum* cv. Taiwan	0.81	0.55
Total	16.06	9.30

^1^ By-product of the orange industry.

**Table 3:** Daily enteric CH_4_ emissions in dry cows and at different stages of lactation by breed group (mean + sd).

Period	F1 ^1^	Triple Cross	Jersey
Non-lactating	202.96 (55.77)	250.19 (43.99)	267.44 (55.34)
Lactation stages:			
<76 days	232.74 (93.24)	350.11 (80.24)	410.30 (140.46)
76–150 days	363.86 (56.02)	385.24 (75.03)	386.48 (100.43)
>150 days	224.77 (51.83)	272.35 (51.37)	222.53 (53.25)
Annual mean	274.49 (24.15)	322.69 (29.49)	297.77 (32.42)

^1^ Breed Groups: F1: 50% Jersey × 50% Gyr; Triple cross: 50% Jersey × 31% Holstein × 19% Sahiwal.

**Table 4:** Annual emission of enteric CH_4_ in cows according to breed group (kg cow ^−1^) (mean + sd).

Period	F1 ^1^	Triple Cross	Jersey
Dry cow	17.25 (2.00)	21.27 (1.88)	22.73 (2.85)
Lactating cow	73.97 (14.78)	90.56 (21.29)	88.69 (22.76)
Annual emission	91.22 (12.78)	111.82 (19.41)	111.42 (19.90)

^1^ Breed Groups: F1: 50% Jersey × 50% Gyr; Triple cross: 50% Jersey × 31% Holstein × 19% Sahiwal.

**Table 5:** Feed consumption, milk production and CH_4_ emissions in lactating and dry cows of different breed groups (mean + sd).

	Lactating Cows				Dry Cows			
Variables	F1 ^1^	Triple Cross	Jersey	Mean	F1	Triple Cross	Jersey	Mean
Total DMI (%BW)	3.54 (0.17) ^a^	3.71 (0.19) ^a^	3.91 (0.17) ^a^	3.69 (0.07) ^a^	1.89 (0.11) ^a^	1.86 (0.12) ^a^	2.19 (0.12) ^a^	2.02 (0.09) ^b^
Grass DMI (%BW)	1.59 (0.01) ^a^	1.56 (0.01) ^a^	1.56 (0.01) ^a^	1.57 (0.01) ^b^	1.75 (0.03) ^a^	1.75 (0.03) ^a^	1.74 (0.03) ^a^	1.75 (0.01) ^a^
Production of milk (kg cow^−1^ day^−1^)	18.19 (1.78) ^a^	18.25 (1.93) ^a^	17.77 (1.87) ^a^	18.17 (0.57)				
CH_4_ emission (g kg^−1^ of milk)	16.09 (4.41) ^a^	17.38 (4.65) ^a^	18.76 (5.71) ^a^	16.95 (1.15)				
CH_4_ emission (g kg^−1^ DM)	16.71 (4.05) ^a^	19.84 (4.17) ^a^	19.78 (5.54) ^a^	17.82 (1.30) ^b^	26.70 (7.57) ^a^	29.86 (6.4) ^a^	27.08 (7.52) ^a^	29.40 (1.87) ^a^

^a,b^ Means with different letters across rows are significantly different according to Fisher’s LSD test (*p* > 0.05).^1^ Breed Groups: F1: 50% Jersey × 50% Gyr; Triple cross: 50% Jersey × 31% Holstein × 19% Sahiwal.

**Table 6:** Enteric CH_4_ conversion factor (Y_m_—%) in dry and lactating cows according to breed group (mean + sd).

Period	F1 ^1^	Triple Cross	Jersey
Dry	6.9 (1.22) ^b^	9.87 (1.37) ^a^	10.28 (1.3) ^a^
Lactation stages (days)			
<76	4.44 (0.91) ^a^	6.25 (0.98) ^a^	7.25 (1.27) ^a^
76–150	7.11 (1.04) ^a^	7.20 (1.51) ^a^	6.91 (1.63) ^a^
>150	5.14 (0.72) ^a^	5.55 (0.84) ^a^	3.79 (0.79) ^a^
Mean	5.90 (0.58) ^a^	7.22 (0.69) ^a^	7.05 (0.73) ^a^

^a,b^ Means with different letters across rows are significantly different according to Fisher’s LSD test (*p* > 0.05).^1^ Breed Groups: F1: 50% Jersey × 50% Gyr; Triple cross: 50% Jersey × 31% Holstein × 19% Sahiwal.

## Data Availability

The digital dataset of the present study is available from the corresponding author upon reasonable request.
